# Sensitivity of Pressure Sensors Enhanced by Doping Silver Nanowires

**DOI:** 10.3390/s140609889

**Published:** 2014-06-04

**Authors:** Baozhang Li, Chengyi Xu, Jianming Zheng, Chunye Xu

**Affiliations:** CAS Key Laboratory of Soft Matter Chemistry, Department of Polymer Science and Engineering, Hefei National Laboratory for Physical Sciences at the Microscale, University of Science and Technology of China, Hefei 230026, China; E-Mails: bzlee@mail.ustc.edu.cn (B.L.); xuchyi@mail.ustc.edu.cn (C.X.); jmz@ustc.edu.cn (J.Z.)

**Keywords:** PVDF, electrospun, pressure sensor, piezoelectricity, β phase, flexible, high performance

## Abstract

We have developed a highly sensitive flexible pressure sensor based on a piezopolymer and silver nanowires (AgNWs) composite. The composite nanofiber webs are made by electrospinning mixed solutions of poly(inylidene fluoride) (PVDF) and Ag NWs in a cosolvent mixture of dimethyl formamide and acetone. The diameter of the fibers ranges from 200 nm to 500 nm, as demonstrated by SEM images. FTIR and XRD results reveal that doping Ag NWs into PVDF greatly enhances the content of β phase in PVDF. This β phase increase can be attributed to interactions between the Ag NWs and the PVDF matrix, which forces the polymer chains to be embedded into the β phase crystalline. The sensitivity of the pressure sensors agrees well with the FTIR and XRD characteristics. In our experiments, the measured sensitivity reached up to 30 pC/N for the nanofiber webs containing 1.5 wt% Ag NWs, which is close to that of poly(vinylidene fluoride-trifluoroethylene) [P(VDF-TrFE), (77/23)]. This study may provide a new method of fabricating high performance flexible sensors at relatively low cost compared with sensors based on [P(VDF-TrFE), (77/23)].

## 1. Introduction

PVDF is well known for its outstanding ferroelectrical [1], pyroelectrical [2], and piezoelectrical [3] properties. It is widely used in human-related applications for its flexibility, light weight and biocompatibility, e.g., in sphygmomanometers [4], pressure sensors [5–8] and nanogenerators [9]. It is a semicrystalline material with a nonpolar crystalline α phase (*trans*-gauche conformation, TGTG′), crystalline polar β phase (all *trans*-conformation, TTT), crystalline γ phase (one gauge four units, T_3_GT_3_G′) and crystalline δ phase. The crystalline β phase is an attractive structure as s polar crystalline form in PVDF, while the crystalline γ phase [10] and crystalline δ phase have poor properties compared with the crystalline polar β phase. The piezoelectric response of PVDF originates from the deformation of the crystalline polar β phase under the exerted pressure, in which the C-F dipoles arrange in the same direction. The polymer chains in the crystalline β phase stretch in the TTT conformation with the dipoles aligning in the direction perpendicular to the polymer chains. However, in pristine PVDF, polymer chains are tangled and tend to form a random coil structure with the TGTG′ conformation, which easily to pile up into the crystalline nonpolar α phase. Since the crystalline polar β phase contributes to piezoelectric signals, many more publications have been reported about transforming the crystalline α phase into crystalline β phase by elongating the polymer chains to exhibit TTT conformation. Different methods were put into practice such as applying a high electric field, mechanical stretching [11,12], electrospinning [13,14] and doping [15–17]. Kawai [3] reported electric field polarized PVDF whereby the piezoelectricity of PVDF was first discovered. Sajkiewicz [11] and Heymans [12] studied the effect of mechanical stretching processes on the piezoelectricity of PVDF. Doshi [13] and Zhao [14] processed PVDF films produced via electrospinning which showed higher crystalline β phase content than produced by the common casting and spinning coating methods. Attempts have also been made to induce crystalline polar β phase formation in PVDF by doping chemical agents. Boey [15] and Mandal [16] prepared PVDF composites by adding carbon nanotubes into PVDF, which showed that the composite has a higher crystalline β phase content. However, carbon nanotubes are hard to disperse and cost a lot. Lee [17] obtained composites with a high content of crystalline β phase by doping palladium nanoparticles into PVDF. These results may be attributed to local-dipole effects which force the C-F dipoles on PVDF chains to oriente and form the crystalline β phase on the surfaces of the dopants. However, in their work they did not measure the piezoelectricity of the obtained composites.

Recently, silver nanowires (Ag NWs), a typical one-dimensional material with lengths of up to tenths of micrometers, has attracted much attention for its high aspect ratio [18], easy synthesis and high electrical conductivity [19] (in line with bulk silver). Ag NWs have several advantages when serving as the filler in a polymer matrix. Doping Ag NWs could greatly enhance surface enhanced Raman scattering performance and the conductivity of Ag NW composite electrodes [20], so the low cost and good dispersion properties make Ag NWs a more attractive candidate as dopant in PVDF.

Our previous publications have focused on the processing method and properties of electrospun PVDF and P(VDF-TrFE) nanofiber webs [5,6,8]. In this paper, we aimed to enhance the crystalline β phase content in PVDF by combining Ag NWs dopant with the electrospinning technology. It turns out that Ag NWs doped PVDF nanofiber webs have higher content of crystalline β phase and lower content of crystalline α phase than pure PVDF nanofiber webs. The Ag NWs also have a good dispersion in PVDF matrix according to SEM and TEM images. What's more, pressure sensors based on the composites were fabricated and measured by methods which have been publicized previously.

## 2. Experimental Section

### 2.1. Materials

Poly(vinylidene fluoride) (Mn = 543,600) was from Sigma Aldrich (St. Louis, MO, USA), Polyvinylpyrrolidone (PVP), silver nitrate (AgNO_3_), silver chloride (AgCl), ethylene glycol (EG), acetone and N,N-dimethylformamide (DMF) were purchased from Sinopharm Chemical Reagent Co., Ltd. (Shanghai, China). The Ag NWs was synthesized using a method previously reported in [19]. All the materials were used without further treatment.

### 2.2. Electrospinning Process

Viscous electrospinning solutions were prepared by dissolving PVDF (1.5 g) and Ag NWs fillers in a cosolvent mixture of DMF and acetone (10 mL, weight ratio 2:3). The solutions were stirred and sonicated at 50 °C for 2 h. To figure out the effect of Ag NWs concentration on the sensitivities of the webs, four different electrospinning solutions were prepared with Ag NWs concentrations of 0, 0.5, 1.5 and 3.0 wt%. The homogeneous solutions were transferred into 20 mL syringes. A stainless flat-end steel needle was used to feed the solutions. A grounded copper plate covered with aluminum foil was used as collector, which was kept 15 cm away from needle tip. The electrospinning process was conducted at a high voltage of 12 kV. The feed rate was fixed at 1 mL/h by using a flow-metering pump. The atmospheric temperature was kept steady at 25 °C and the humidity was 32%. A smooth and breathable film with a thickness of about 100 μm was obtained from the collector. The area of the film corresponded with the collector and was about 400 cm^2^.

### 2.3. Characterizations

A nanofiber electrospinning unit (NEU-010; KES Kato Tech Co., Ltd., Kyoto, Japan) was used to produce nanofiber webs. Surface morphologies of the nanofiber webs were examined with a scanning electron microscope (SEM, Sirion 200; FEI, Hillsboro, OR, USA). All the samples were sputtered with gold before testing. Field-emission transmission electron microscopy (FETEM) was performed on a JEM-2100F instrument (JEOL, Tokyo, Japan) to study the inner structures of fibers. The samples were prepared by directly electrospinning the fibers on special copper grids for TEM analysis. X-ray diffraction spectrometry (TTRAX III; Rigaku, Tokyo, Japan) and unpolarized attenuated total reflectance Fourier transform infrared spectroscopy (ATR-FTIR, Nicolet 8700; Thermo Fisher Scientific Inc., Waltham, MA, USA) were used to identify the polycrystalline structures of the as-electrospun nanofiber webs. The sensitivities of the nanofiber webs were characterized using a homemade setup, which was reported in our previous work [5,6]. The interactions between polymer chains and Ag NWs surfaces were characterized using UV-visible spectrophotometry (JASCO V-670, City Tokyo, Japan) and FTIR.

## 3. Results and Discussion

### 3.1. Surface Morphology

**Figure 1.**
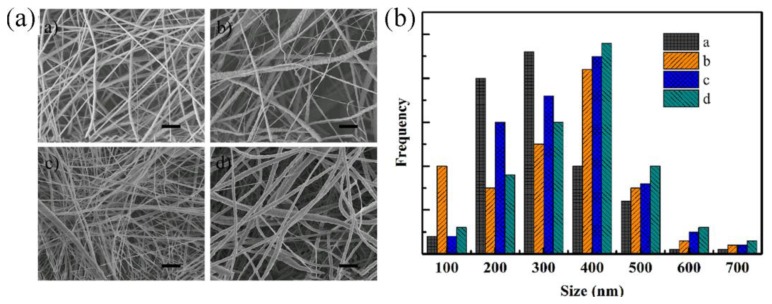
SEM images (**a**) and diameter distribution of the as-electrospun nanofiber (**b**). Naonofiber webs of (a) pure PVDF; (b) 0.5 wt% Ag NWs doped PVDF; (c) 1.5 wt% Ag NWs doped PVDF; (d) 3.0 wt% Ag NWs doped PVDF. The length of the bars inserted in the picture is 1 μm.

Figure 1a is typical SEM micrograph of nanofiber webs made by our electrospinning method. The as-electrospun nanofiber webs consist of randomly oriented nanofibers. The diameters of the fibers (Figure 1b) mainly range from 200 nm to 500 nm with only few beads on the fibers. With more Ag NWs doped into PVDF nanofibers, the surface of fibers become rougher. The reason may be the fact that dielectric constant of the electrospinning solutions, which affects the charge distribution on the surface of the fiber while the mixture solution are electrospun, were changed after doping Ag NWs into PVDF. No isolated Ag NWs were found in the SEM images, indicating the Ag NWs are well dispersed into the fibers. Additionally, the diameters of the fiber have a slight increase when the Ag NW concentration varies from zero to 3 wt%.

### 3.2. Polymorphism Structures of Nanofiber Webs

**Figure 2.**
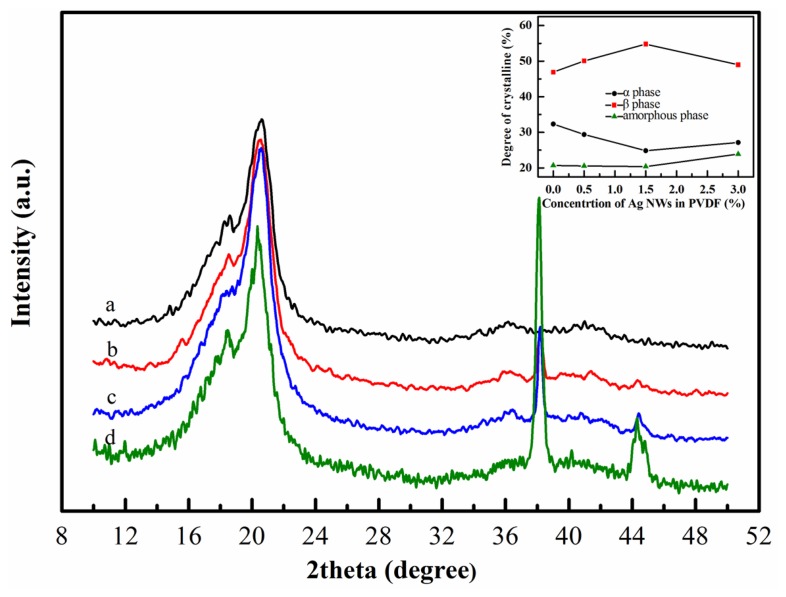
XRD patterns of the electrospun nanofiber webs with the concentration of Ag NWs doped: (**a**) 0. (**b**) 0.5 wt%. (**c**) 1.5 wt%. (**d**) 3.0 wt%.

**Figure 3.**
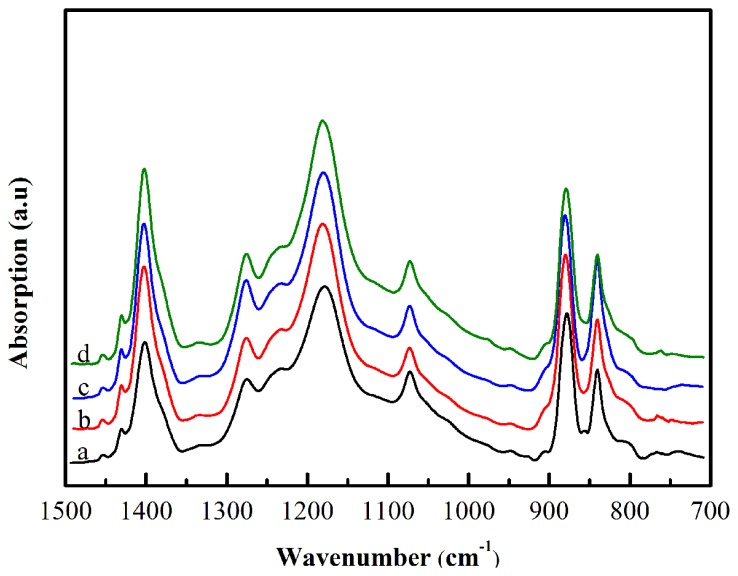
ATR-FTIR analysis of electrospun nanofiber webs. The curves in this graph represent (**a**) 0. (**b**) 0.5 wt%. (**c**) 1.5 wt%. (**d**) 3.0 wt% Ag NWs doped PVDF nanofiber web, respectively.

The X-ray diffraction curves of nanofiber webs are shown in Figure 2. The sharp diffraction peaks at the 2θ value of 38° and 44° present the (111) and (200) reflection planes of Ag NWs, respectively. For pure PVDF nanofiber web (curve a in Figure 2), a strong and broad diffraction peak at the 2θ value of 20.6° corresponds to (200) and (110) reflection planes of the crystalline β phase. The shoulder peak at the 2θ value of about 18.2° corresponds to (020) and (100) planes of crystalline α phase. In addition, the distinctive peak at the 2θ = 36.5° correspond to crystalline β phase. For the curves of the nanofiber webs doped by Ag NWs (curves b, c, d in Figure 2), the diffraction peak of crystalline β phase at 2θ = 20.6° rise and the diffraction peak of crystalline α phase at 2θ = 18.2° weaken compared with that of pure PVDF. The ATR-FTIR spectrums of the nanofiber webs (Figure 3) corroborate the X-ray diffraction results. The absorption peaks at 1276 and 839 cm^−1^ are typical vibration characteristics of crystalline β phase, where the intensities increase after doping Ag NWs into PVDF, while the intensities of the absorption peaks at 977, 796 and 762 cm^−1^, which are attributed to the crystalline α phase, drop after doping Ag NWs into PVDF.

It is notable that doping Ag NWs into PVDF has a distinctive effect of both enhancing the crystalline β phase and weakening the crystalline α phase. This effect is closely related to the concentration of Ag NWs. For the spectrum of 0.5 wt% Ag NWs doped PVDF (curve b in Figure 3), the intensities of the crystalline β phase absorption bands at 1276 and 839 cm^−1^ improve compared with that of pure PVDF (curve a in Figure 3). The improvement remains as the concentration of the Ag NWs reaches up to 1.5 wt%. However, when the concentration of the Ag NWs is 3.0 wt%, it becomes complicated. As indicated by curve d in Figure 3, the intensities of the absorption bands at 1276 and 839 cm^−1^ decrease and those at 796 and 762 cm^−1^ increase compared with that of 1.5 wt% Ag NWs doped PVDF. Since Ag NWs have an electron-rich surface, C-F bonds on the polymer chain are electron-deficient groups. The UV-Vis spectrum and ATR-FTIR (Figure 7) indicate the interactions between Ag NWs and PVDF, which force the polymer chain to expand in a TTT conformation and overlie on the surface of Ag NWs. This local *trans* conformation may facilitate the nucleation of a polar β phase, which was confirmed in other studies [10,17]. However, the initial α phase nucleation is hard to form in the bulk matrix since they are induced by the thermal motion of polymer chains. Also, adding Ag NWs prevents TGTG conformation (α crystalline form). Nevertheless, when the concentration of Ag NWs increases to 3.0 wt%, too many β phase spherulites form and tend to squeeze together, so crystalline α phase is likely to form in the inter-region between spherulites corresponding to an amorphous-like phase, which also was discovered by Drzal's group who doped exfoliated graphite into polypropylene [21]. It is proved that the amorphous phase and crystalline α phase increase from the insert graph in Figure 2. The mechanism of β phase enhancement may be similar to the effect of exfoliated graphite doped into polypropylene [21] and palladium nanoparticles doped into PVDF [17], where the dopants not only act as a crystal nucleus but also affect crystal structure of the matrix. Furthermore, the X-ray diffraction results (Figure 2) are in accordance with the FTIR analysis (Figure 3) in that the composite of 1.5 wt% Ag NWs doped PVDF nanofiber web is observed to possess the most crystalline β phase and the least crystalline α phase. Furthermore, the nanofiber web is obtained using electrospinning whereby a huge DC voltage is applied between the tip of the syringe and the collector. The β phase is preferentially polarized through the DC voltage, which was ascertained by Kim's group [22].

### 3.3. Sensitivity of the Nanofiber Webs

**Figure 4.**
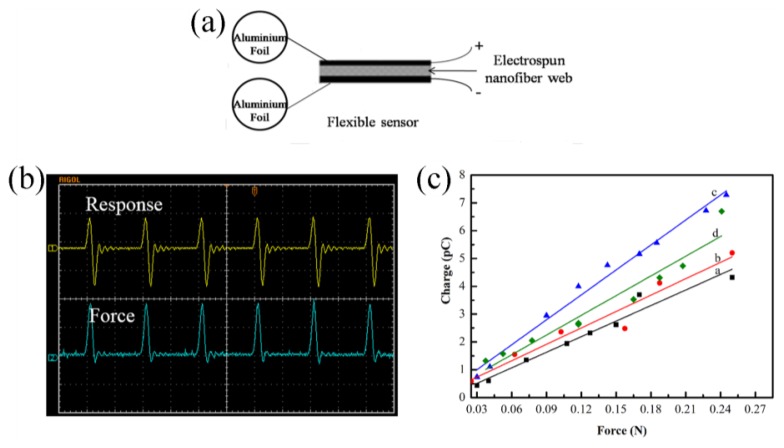
The schematic of the device (**a**) and response of the pressure sensors recorded by oscilloscope (**b**). Plotted line of the response to the exerted force (**c**); a: pure PVDF, b: 0.5 wt% Ag NWs doped PVDF nanofiber web, c: 1.5 wt% Ag NWs doped PVDF nanofiber web, d: 3.0 wt% Ag NWs doped PVDF nanofiber web.

The sensitivity of our device is related to the content and polarization of the β phase in the polymer matrix. Equipment and methods, reported in our previous publications [5,6], are used to test the sensitivities of the obtained nanofiber webs. The pressure sensors (Figure 4a) are assembled by laminating two pieces of aluminum foil to the sides of the nanofiber webs. The effective area of the devices (5 mm × 5 mm) is about 25 mm^2^. We used an oscilloscope to record the response signals (Figure 4b) of exerting forces and output charges of the pressure sensors, which are plotted in Figure 4c). It reveals that the output signals have a good linear relationship with external forces. The sensitivities of the sensors listed in Table 1 are inferred from the slopes. Consistent with polymorphism analysis, the pressure sensor (1.5 wt% doped Ag NWs) shows the highest sensitivity, up to 29.8 pC/N. The pressure sensors doped with 0.5 wt% and 3.0 wt% Ag NWs exhibit 19.7 pC/N and 23.8 pC/N, respectively. A sensitivity of only 18 pC/N is observed from the pressure sensor based on pure PVDF. This demonstrates that the combination of doping Ag NWs into PVDF matrix and electrospinning can greatly improve the sensitivity of PVDF.

### 3.4. TEM Images of the Nanofiber Webs

**Figure 5.**
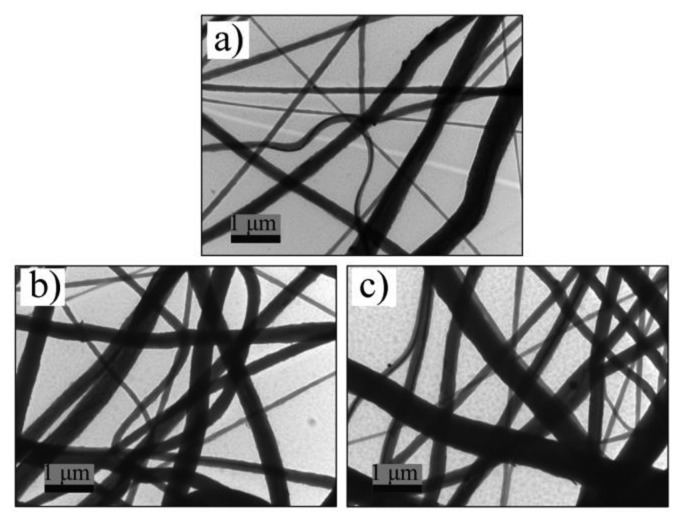
TEM micrographs of Ag NWs doped PVDF. 0.5% Ag NWs doped nanofiers (**a**). 1.5% Ag NWs doped nanofibers (**b**). 3% Ag NWs doped nanofibers (**c**).

**Figure 6.**
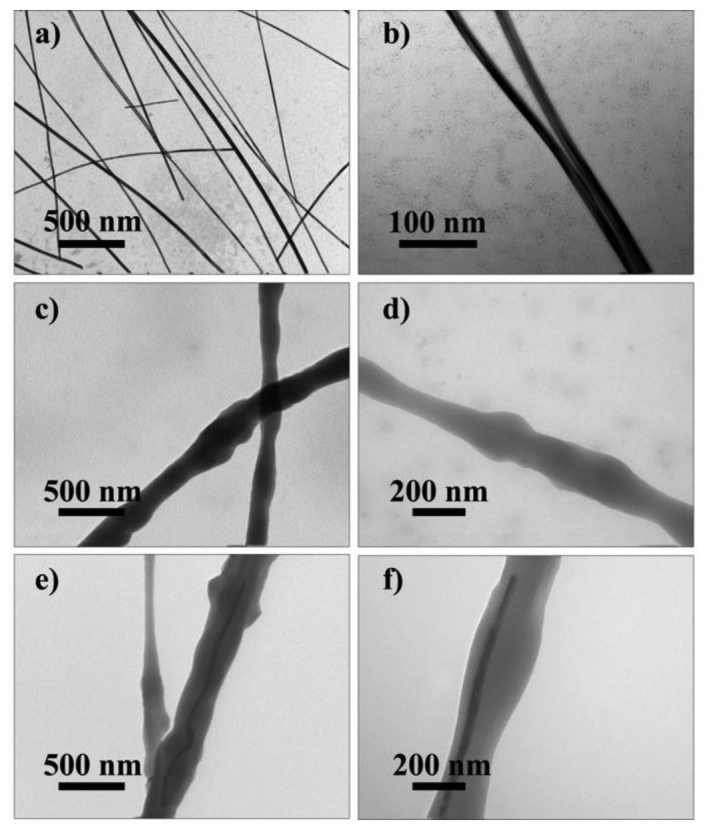
EM micrographs of Ag NWs (**a**, **b**). nanofibers of pure PVDF (**c**, **d**). nanofibers of 1.5 wt% Ag NWs doped PVDF (**e**, **f**).

Moreover, TEM images (Figures 5 and 6) were taken to investigate the inner structure of the nanofibers The Ag NWs have smooth surfaces with the diameter of 40 nm (a, b in Figure 6). To further investigate that Ag NWs are embedded into PVDF fibers rather than separated from PVDF matrix, samples made of pure PVDF and Ag NWs doped PVDF are tested. The results presented in Figure 6c–f prove that the Ag NWs doped in PVDF are inserted into the fibers rather than isolated from the PVDF matrix, which indicates the compatibility of Ag NWs and PVDF matrix. Besides, the diameter of fibers in TEM images (Figure 6c–f) identify with that in the SEM images (Figure 1). The Ag NWs are individually dispersed in the PVDF matrix which shows they are well-dispersed in the PVDF matrix.

### 3.5. Ag NWs Induced β Phase

**Figure 7.**
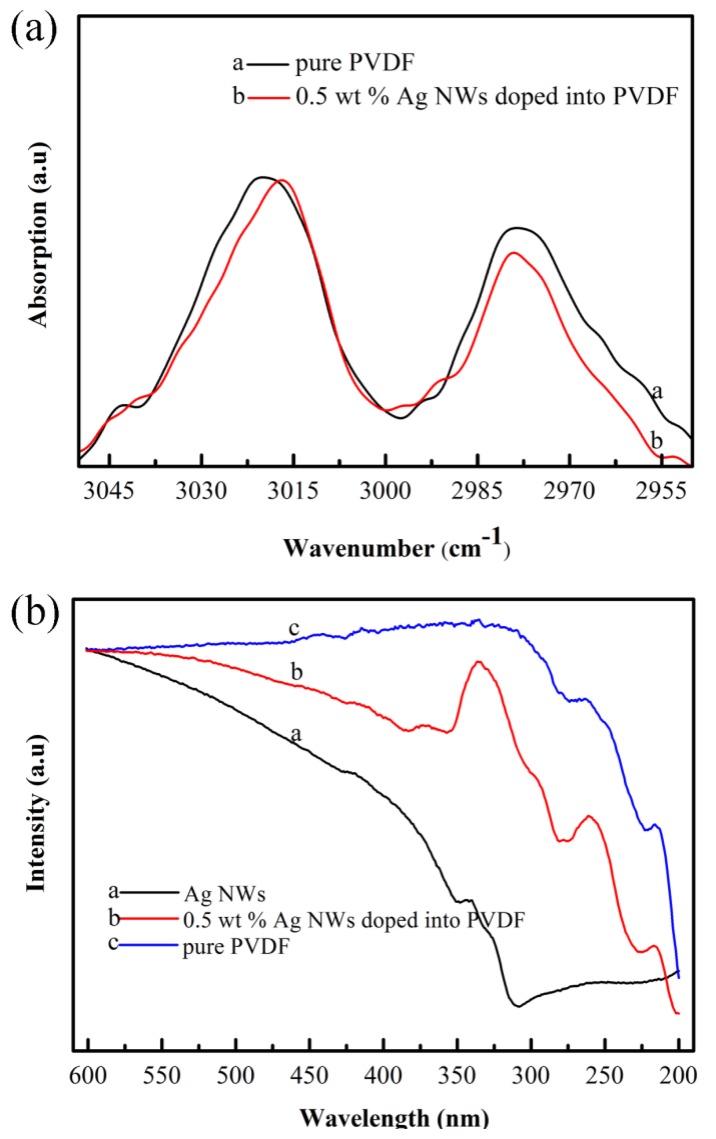
Characteristics of the interactions between PVDF matrix and the Ag NWs surfaces according to UV-Vis spectra (**a**) and ATR-FTIR (**b**).

The interactions between PVDF matrix and Ag NWs are characterized using UV-Vis spectrophotometry and ATR-FTIR (Figure 7). For the UV-Vis spectra (Figure 7a) of the nanofiber webs, the absorption peaks at 219 nm and 260 nm are attributed to PVDF. The absorption peaks of Ag NWs are located at 324 nm and 341 nm. Comparing curves a and b in Figure 6a, it is found that the absorption peaks of Ag NWs doped into PVDF nanofiber webs have a slight red shift, which indicates the interactions between the polymer chains and Ag NWs surfaces. Also the FTIR results (Figure 7b) show a 3 cm^−1^ red shift of the absorption bands at 3021 cm^−1^ which represents the asymmetrical stretching vibration of C-H bond. Incorporating the TEM result (in Figure 6), they prove the previous hypothesis that the Ag NWs are beneficial for forming the initial β crystal nucleus. Metal nanowires such as Ag NWs, have electron-rich surfaces. Thus the local-dipole field of the electron-rich surfaces impel polymer chain to exhibit a TTT conformation, causing the polymer chains to align on the surfaces of Ag NWs and form crystalline polar β phases.

## 4. Conclusions

We have fabricated and investigated novel flexible pressure sensors based on the composites doped with different concentrations of Ag NWs. It is observed that Ag NWs are well dispersed in PVDF. The UV-Vis and ATR-FTIR spectra imply that interactions between Ag NWs and PVDF chains give rise to crystalline β phase enhancement. This can be explained by the fact that the surface of Ag NWs acts as a initial β phase crystal nucleus which makes the TTT conformation stable and the crystalline β phase easy to form. However, it turns out that the content of crystalline polar β phase in composites doped 3.0 wt% Ag NWs is less than that of 1.5 wt%. The best sensor tested is fabricated using nanofiber webs of electrospun PVDF doped with 1.5 wt% Ag NWs, whose sensitivity reaches up to 30 pC/N. This performance could match that of P(VDF-TrFE) (77/23) nanofiber web [5] but with a much lower cost. Consequently, this research provides an effective way to develop an efficient flexible and economical piezoelectric material.

## Figures and Tables

**Table 1.** Sensitivities of the pressure sensors based on nanofiber webs.

**Nanofiber Webs (Content of Ag NWs)**	**Sensitivity (pC/N)**	**Standard Error (R^2^)**
0	18.1	0.96
0.5%	19.7	0.93
1.5%	29.8	0.98
3.0%	23.8	0.97
